# Human resources and the quality of emergency obstetric care in developing countries: a systematic review of the literature

**DOI:** 10.1186/1478-4491-7-7

**Published:** 2009-02-06

**Authors:** Maman Dogba, Pierre Fournier

**Affiliations:** 1 Département de santé publique, Université de Montréal, Montréal, Québec, Canada; 2 Unité de santé internationale, Université de Montréal, Montréal, Québec, Canada

## Abstract

**Background:**

This paper reports on a systematic literature review exploring the importance of human resources in the quality of emergency obstetric care and thus in the reduction of maternal deaths.

**Methods:**

A systematic search of two electronic databases (ISI Web of Science and MEDLINE) was conducted, based on the following key words "quality obstetric* care" OR "pregnancy complications OR emergency obstetric* care OR maternal mortality" AND "quality health care OR quality care" AND "developing countries. Relevant papers were analysed according to three customary components of emergency obstetric care: structure, process and results.

**Results:**

This review leads to three main conclusions: (1) staff shortages are a major obstacle to providing good quality EmOC; (2) women are often dissatisfied with the care they receive during childbirth; and (3) the technical quality of EmOC has not been adequately studied. The first two conclusions provide lessons to consider when formulating EmOC policies, while the third point is an area where more knowledge is needed.

## Introduction

Of the estimated 529 000 annual maternal deaths worldwide, 99% occur in developing countries, making maternal mortality a major health and development challenge. Among women who avoid maternal death, approximately 10 million suffer from complications related to pregnancy and childbirth [1,2]. Maternal mortality is therefore both a health and a development indicator. In fact, the risk of dying during pregnancy is 1/6 in the poorest countries compared with 1/30 000 in Northern Europe [2]. Because of the magnitude and negative consequences of maternal mortality, its reduction has mobilized the international community. The fifth Millennium Development Goal is to reduce maternal mortality by 75% between 1990 and 2015 [3]. Recent evaluations show that progress has been especially slow in sub-Saharan Africa because of weakened health systems, poor quality of care, inadequate human resources, financial barriers to care and insufficient political commitment [4-7].

Most maternal deaths are avoidable. They are the result of major direct obstetric complications (haemorrhage, uterine rupture, dystocia, eclampsia) and indirect complications (HIV, malaria) [2]. Most direct obstetric complications can be treated by a package of eight interventions identified by the World Health Organization (WHO), the United Nations Children's Fund (UNICEF) and the United Nations Population Fund (UNFPA) that, taken together, are known as emergency obstetric care (EmOC):

1. parenteral antibiotics;

2. parenteral oxytocic drugs;

3. parenteral anticonvulsants for pregnancy-induced hypertension;

4. manual removal of the placenta;

5. removal of retained products of conception;

6. assisted vaginal delivery;

7. surgery (e.g. caesarean delivery);

8. blood transfusion.

Health facilities that provide the first six interventions are called basic EmOC centres, as compared to complete EmOC centres that can provide all eight [5,8,9].

Though the clinical techniques for combating maternal death and morbidity are well known, choosing the best strategies to implement remains a huge challenge for developing countries [10]. Historical analyses show that declines in rates of maternal mortality result from a combined effect of several technical and political factors. No single strategy is effective at significantly lowering the rate of maternal mortality [11].

Skilled birth attendance and emergency obstetric care are two recent strategies promoted to reduce maternal mortality [2,9]. Yet, even if the capacity to supply EmOC is the minimum starting point, it must be coupled with strategies to reduce delays in receiving care and to increase care coverage.

Therefore, the intrapartum health centre strategy constitutes, to date, the combination of interventions best suited to produce significant declines in maternal mortality rates [12]. This strategy is not restricted to women presenting complications, but targets all women during the childbirth period.

However, to ensure that every woman receives skilled care at childbirth in an appropriate environment is clearly a "respectable but distant" objective due to the limited resources of many developing countries [13]. Indeed, in many rural regions of developing countries, deliveries are still handled by traditional birth attendants. In such contexts, a better coverage of obstetric emergencies can help lower the still very high maternal mortality rates.

The intrapartum health centre strategy aims at ensuring deliveries in health centres with midwives and their assistants. These qualified personnel are able to provide adequate essential obstetric care to women. However, they must also be able to detect complications and handle them, either by giving basic EmOC or by referring the most complicated cases to well-equipped hospitals for complete EmOC.

Even when the best combinations are identified, many obstacles must still be overcome. Among them is the inadequacy of human resources (HR) in developing countries. In the health sector in general, and in maternal health in particular, health care professionals are at the heart of the success of EmOC interventions [13]. The performance of any health system, and thus the improvement of a population's health, depends on the productivity, competence, availability and responsiveness of health professionals [14].

In maternal health, as reported by historical analyses, professionalization of midwives is among the successful HR strategies that have contributed to reducing maternal mortality in developed countries [11]. Conversely, the promotion of traditional birth attendance has been one of the recently promoted HR-centred strategies that has failed to reduce maternal mortality significantly in developing countries.

The intrapartum health centre strategy relies on sufficient coverage of good-quality EmOC and on a functional reference centre. EmOC services are excellent markers for monitoring and measuring health system performance. Variations in their quality are rapidly expressed as changes in measurable outcomes such as maternal and infant mortality. Moreover, the technical nature of EmOC and the necessary interaction between patients and professionals during care delivery are such that HR occupies a pivotal position in EmOC. Thus, to ensure good-quality care, one of the major obstacles to be overcome is HR inadequacy.

What is known about the role of HR in providing quality EmOC? And how does the available knowledge translate into health policies? To answer these questions, we performed a literature review to determine the role of HR in quality EmOC, to collect available evidence and to identify knowledge gaps about HR performance. Our ultimate goal is to inspire current and future studies and policies for EmOC quality improvement that focus on the role of HR. It should be noted that the role of HR in referrals to higher-level facilities has not been treated here, as it would require a separate literature review.

## Materials and methods

### Data sources and search strategy

In March, 2007, we performed a search of two electronic databases: ISI Web of Science (1979 to March 2007) and MEDLINE (1950 to March 2007). The following combination of keywords was used: "quality obstetric* care" OR "pregnancy complications OR emergency obstetric* care OR maternal mortality" AND "quality health care OR quality care" AND "developing countries".

Based on the advice of one of the authors (PF), an expert in the field of maternal health and EmOC, the search strategy was supplemented by a systematic review of the table of contents of specific targeted journals: *International Journal of Gynaecology and Obstetrics*, *Reproductive Health Matters *and *The Lancet*. In consulting the first studies on EmOC in the literature, this screening was limited to the period from 1990 to 2007. EndNote 9 software was used for reference management.

### Study selection

Our eligibility criteria for selecting articles were that they were either quantitative or qualitative empirical studies on the quality of EmOC in developing countries. No restrictions on the term "quality" were established a priori. Although there was no language restriction in the search criteria, only studies published in English and French were selected. The grey literature was not consulted. Letters, editorials, comments and opinions were excluded. Additionally, studies carried out in developed countries and articles that addressed the quality of maternal care in general, the quality of health systems, or traditional birth attendants were not included.

A two-stage selection process was used. First, articles were retained based on their titles, keywords and summaries. Retained articles were then analysed in depth and their reference lists carefully screened. Supplementary studies responding to the above criteria were thus identified, as in a "snowball" approach.

### Data extraction and synthesis

We did not aim to perform a meta-analysis (quantitative description of the literature); therefore, we did not perform a quantitative rating of evidence power. Rather, we carried out a narrative synthesis and a descriptive summary of the selected studies. These studies were classified qualitatively, on a decreasing hierarchical basis, as follows: systematic or narrative literature reviews; explanatory analytical studies; normative evaluation studies; and descriptive articles. Based on an assessment of each component of the intervention against norms and criteria, normative evaluation studies compare the observed effects with the desired effects of the intervention [15,16].

We devised a data extraction form to categorize the selected articles. This form specified the types of studies, their objectives, methodologies, locations and key results. Once the relevant data had been extracted and the studies summarized, the remainder of the analysis was carried out using the data extraction form and the analytical framework presented in the following section.

### Analytical framework

**Figure 1:**
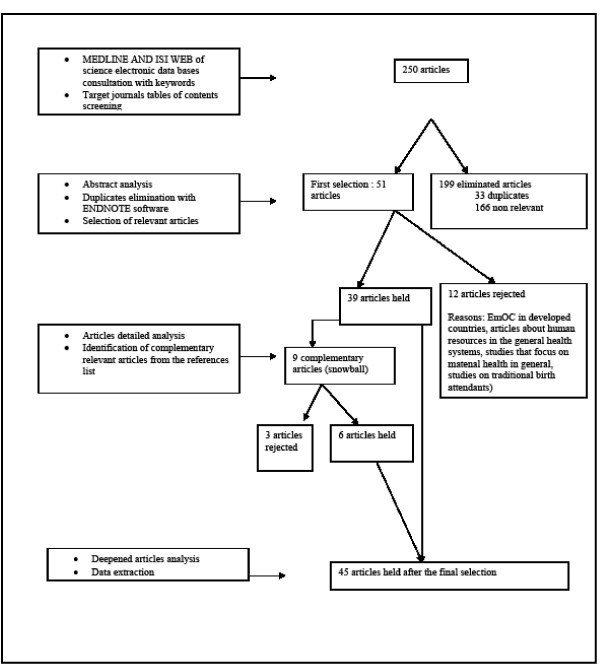
**Analytical framework and data analysis results**.

The concept of "quality of care" was defined in this study as the level at which health services increase the probability of the desired results for individuals and populations, according to the current state of knowledge [17]. From this definition, we were able to conceptualize EmOC into three customary dimensions of quality: structure, process and results [18-20]. Structure includes material, human and organizational resources. Process includes the clinical, technical and interpersonal aspects of care. Results include maternal and newborn health indicators and the users' assessment of care [18-20]. This framework [21] was modified and adapted to support the analysis of the above-mentioned dimensions of EmOC quality. The results are reported in Fig. 1.

As is seen in Fig. 1, HR components can be identified in each of the three dimensions of EmOC quality. The structural dimension of care includes, besides the HR component, organizational and material resources components. The process dimension is essentially made up of HR, in terms of technical quality, interpersonal quality and motivation with respect to EmOC.

The different categories of quality of care used to classify studies are not mutually exclusive; a given study can be classified in several categories. However, for each selected study, its main objective or core question allowed us to identify a central theme. When secondary objectives were clearly specified in the selected studies, or when results touched upon themes that were different from the main objective, these aspects were considered to have been partially studied.

### Management of divergent opinions

The search for articles was essentially carried out by the primary author (MD). The selection of articles and their summaries and classification were finalized with the approval of the second author, a senior investigator in maternal health. Divergent opinions were resolved by agreement between the two authors.

## Results

**Figure 2:**
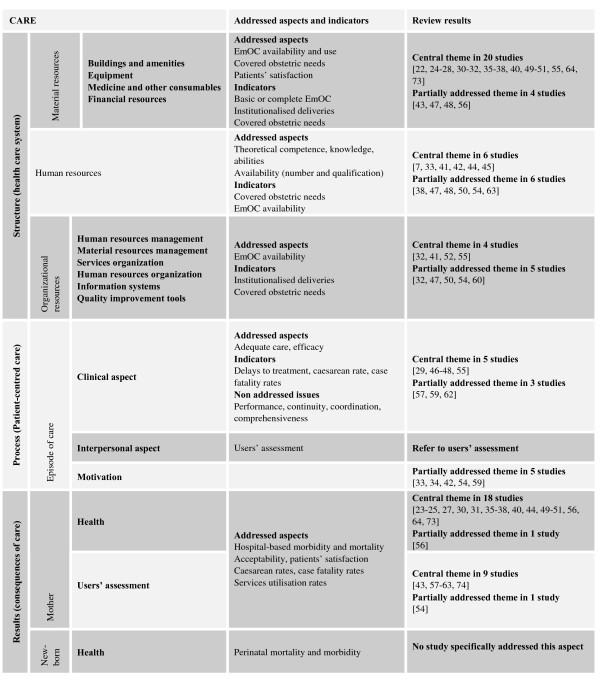
**Review methodology and quantitative overviews**.

Of the 250 articles that met our criteria, 45 were retained for further analysis. Figure 2 presents the various stages of this literature review and their results. Articles finally selected included two literature reviews, seven explanatory analytical studies, six descriptions of EmOC programs, 21 normative evaluations and nine case studies.

In all, 30 articles were classified in the "structure" section, five in "process" and 27 in "results". Most articles addressed several items. Twenty discussed material resources; six, human resources; and four, organizational resources. The structural aspects of EmOC and the interpersonal constituents of the EmOC process were easily identified. The clinical aspects of EmOC, where the role of HR is theoretically essential, were difficult to assess separately from material and organizational resources.

An overview of the studies on EmOC shows that there are many more dealing with the structure of care, and their results are relatively more abundant than those dealing with the EmOC process, the primary component of which is health personnel. Among studies on EmOC structures, material resources are more often evaluated than human and organizational resources.

### EmOC structure

#### Material resources

Most of the interventions that make up EmOC, such as parenteral administration of antibiotics, caesarean delivery, etc., require specific material resources. Depending on their complexity, these interventions are classified as basic EmOC or complete EmOC [5,9]. The availabilities of basic and complete EmOC were assessed by means of specific tools such as the "room by room walk-through" [22], which described the availability of equipment, buildings and medicines for EmOC interventions. This assessment, led primarily by the Averting Maternal Death and Disability Programme, showed that complete EmOC respected United Nations (UN) standards, while basic EmOC was deficient. The assessment was carried out in Cameroon, Chad, Morocco, Nicaragua, Niger, Rwanda, Sri Lanka and Tanzania [22-35].

Key interventions that were most often absent included assisted vaginal delivery and manual removal of the placenta. Among the explanations offered for these clinical deficiencies were limited task delegation to peripheral sector staff, inadequacy of equipment and the absence of a well-equipped unit [29]. Improvements in EmOC supply often increase its utilization, particularly when the community is mobilized and sensitized to its availability [36-40].

#### Human resources

Several HR aspects of EmOC structure are reported in the selected studies, i.e. availability, qualifications and competence.

##### HR availability

A shortage of EmOC skilled care providers is reported in countries affected by the burden of maternal mortality [7,41,42]. The *World health report 2005 *estimated that, over the next decade, 334 000 supplementary midwives or nurse-midwives, 140 000 midwives or nurses and 27 000 doctors and technicians must be trained or retrained. [41]. The selected studies mention several threats, such as immigration, HIV-AIDS and abandonment of public structures that affect the availability of HR for EmOC. They point out that these staff shortages weaken the quality of care by increasing professionals' workloads and patients' waiting times and making infection control more difficult [7,41]. More than a mere shortage, a regional imbalance is noticed in EmOC staff distribution, with rural areas being most affected. While the United Nations standard of at least one complete EmOC centre for 500 000 inhabitants is often reached, very few countries have attained four basic EmOC centres for 500 000 inhabitants [28,42,43]. Furthermore, 24-hour EmOC availability is compromised by fluctuations in staff at nights and weekends [43], sometimes due to political insecurity [34].

##### HR qualifications

The skilled professionals of significance to these studies are, according to United Nations references, midwives, nurses, physicians, anaesthetists and obstetricians [42,44,45]. Unskilled staff, such as traditional birth attendants, are sometimes addressed by these studies. Administrative and management personnel are increasingly involved in interventions aimed at improving EmOC quality, such as clinical audits, but they are not systematically considered to be among the EmOC personnel [35,46,47].

Staff qualifications partially determine their capacity to diagnose and handle patients adequately. Thus, in Senegal in 2002, maternal morbidity was significantly better diagnosed and treated by doctors and midwives than by nurses and traditional birth attendants [44].

Human resources' qualifications also influence users' perceptions of the quality of care. This is reported in Tanzania in 2003, where the low rate of utilization of health centres providing EmOC is partially due to the poor perception of quality of care. This bad perception is the consequence of shortfalls in skilled professionals [33].

The results reported above led the authors to recommend that care team composition and deployment should therefore ensure an adequate mix of clinical skills. It is also recommended that quality improvement mechanisms should involve all categories of staff, including managers [7,33,35,42,48].

##### HR competence

The study results confirm that HR qualifications alone do not guarantee competence. As shown in a skill and knowledge evaluation in Benin, Ecuador, Jamaica and Rwanda, EmOC professionals scored only 50% in the required skills. Knowledge was evaluated using multiple-choice questions and skills, by tests on anatomical models [45]. Among the reasons suggested for this gap in theoretical knowledge and skills are inadequate training methods, insufficient practice of learned procedures due to lack of equipment [35], inability to delegate tasks [29,34,45,49], and large variations in clinical protocols [45].

The authors therefore strongly recommend implementing skill-based training approaches supported by regular clinical supervision, as tried by several teams [9,35,42,50,51]. These approaches would not only be more effective, but would also reduce training time [41,52]. It is recommended that the training content should be centred on active treatment of the third phase of labour [28,45] and on interpersonal communication with the patient [53]. Further studies are needed to determine the ideal number of training years, the type of staff to train and the number of technical procedures needed to guarantee skills [7].

#### Organizational resources

Some organizational resources to improve EmOC quality were addressed in the selected studies: HR management policies and their effects on staff attitude, equipment management, information systems and quality improvement mechanisms [47,48,54]. These studies concluded that strengthening managerial skills would help to better coordinate patient care [35,48,54] and that well-updated data collection is a prerequisite for good analysis of EmOC quality. These organizational aspects should be part of EmOC improvement programmes, as prescribed by the studies [35,37,39,40,55]. Concerning service organization, permanently available care and a functional referral system are indispensable to the effectiveness of EmOC [33,49].

#### HR and EmOC process

##### Clinical aspects of care

Studies that addressed the provision of EmOC assessed HR performance by indirect measures. No clinical audit of HR performance to assess EMOC quality was found in the literature. Performance measures are often combined with an analysis of the availability of material and organizational resources. The selected studies identified insufficient patient surveillance and logistic incapacities in many countries, Côte d'Ivoire, Benin and Rwanda. These deficiencies affected the core services of gynaecology-obstetrics as well as related services (blood banks and laboratories). Clinical care was also affected by financial inaccessibility to care because of longer delays before care could be received [47,48]. In a study of two hospitals in Côte d'Ivoire, the median delays in care for patients varied from one to five hours; the greatest part of that delay was attributed to the purchase of therapeutic material by patients and their families [48].

The overall evaluation of professional skills, together with the quality of equipment, management and organizational resources, shows interdependency among all these aspects of good quality EmOC [34,54,55]. Indeed, some EmOC interventions depend on the availability of specific equipment like forceps, vacuums and tensiometers [29,55]. In such cases, the absence of equipment can decrease the probability of accomplishing these functions [55]. However, unexpected positive or negative staff reactions can occur: use of personal tensiometers by midwives [55]; repair of a defective autoclave by nurses and the systematic practice of episiotomies by nurses when lacking oxytocics [34].

Although evaluating staff skills independently of their working conditions is difficult, clinical audits by multidisciplinary teams seem appropriate to distinguish organizational dysfunctions from staff-related problems [46-48]. Therefore, as revealed in one study in Indonesia, clinical audits are more informative than simple mortality rates, which, without detailed analysis, do not provide information about which EmOC aspects to improve [56].

Besides technical and professional evaluations, the clinical aspects of EmOC were evaluated from the patients' point of view. Some women in Bolivia, the Dominican Republic and Uganda questioned the positioning for gynaecological exams and other routine practices such as pubic shaving, systematic enema and episiotomy [54,57,58]. Indeed, these practices contradict certain traditional and cultural representations of the women. According to other women, vaginal examination is likened to sexual intercourse and sometimes experienced as rape, especially when it is practised by several doctors, one after the other [57]. Some authors suggest revising the medical paradigm of childbirth, such as gynaecological positioning [41,59].

##### Interpersonal aspect of care

The interpersonal aspect of the EmOC process was assessed from the users' perspective by satisfaction questionnaires. Women were interviewed during the period from pregnancy to postnatal care, but only data relative to the delivery were extracted for this literature review. Some of these studies that focused on near-misses were of particular use to our review because they examine the emergency context [60,61].

Women's level of satisfaction with the care received varied according to their expectations, social class and educational levels [62]. The intimidating clinical environment limits women's free and spontaneous expression on the quality of EmOC. However, when specifically questioned, women did not hesitate to express a general dissatisfaction [62].

Some women, especially near-misses, showed gratitude to the staff who saved their life [60,61]. For other women, what matters most is a live newborn, which can offset staff misbehaviour [60]. But generally, overall dissatisfaction is reported.

The multiplicity of professionals, especially in public hospitals, who examine women is difficult to accept in many contexts. Reducing the number of professionals for the gynaecological examination and increasing exposure to female personnel is preferred by most women, except in the case of certain interventions, such as caesarean sections. [43,62].

The importance allotted to the technical dimension of care, to the detriment of psychological support, is denounced [58,62]. Women rarely find in modern health centres and hospitals the accompaniment, communication and empathy that they had with traditional midwives [58]. Clinical procedures are often begun without preliminary explanation; furthermore, expressions of pain by the women may be mocked by staff. Certain women experienced physical violence and insults, especially in the public hospitals. Patients also disapproved of either preferential or discriminating attitudes of staff, according to a patient's economic status or social network [54,57,59,60,63].

This poor quality of care and general dissatisfaction influences patients' use of heath services and compliance with treatment. Hospital obstetric care was thus sought only as a last resort [57,58,63]. Overall, the interpersonal interaction was very unsatisfactory for patients.

This general observation led the authors to recommend that, despite the various expectations and the difficulty of harmonizing clinical procedures, access to EmOC, although proven to be effective, should not be promoted at the expense of the quality of the interaction between staff and patient [59]. New patient-centred communication structures could reconcile the different "cultures" of patients and staff and should be implemented [60]. Besides, intensification of the psychological aspect of care could help reduce the risks of overly medicalized childbirth.

##### HR motivation

HR motivation was not addressed as an exclusive research question in the studies summarized, but it appeared to be important in relation to staff availability and EmOC performance improvement. In Bangladesh, despite EmOC training scholarships, few applications were received because of the reluctance to work in rural areas [42]. This apprehension was particularly marked among females, who made up the majority of EmOC staff [34]. Another example of the effect of less-motivated personnel on EmOC quality is reported in the Dominican Republic, where the quality of care was better when the hospital EmOC staff felt less overloaded [54]. Incentives implemented to increase EmOC staff motivation included flexibility in HR management and supervision, improvements in working conditions, institutionalization of a culture of accountability, application of financial incentives and better career planning [33,34,54,59,60].

##### Quality of care results

Studies that addressed results of EmOC quality found that reported institutional mortality rates for deliveries were above the recommended rate of 1%, while the covered needs and rates of caesareans are below the United Nations-recommended targets [23-27,29,56,64]. A covered needs rate of 100% is a good EmOC quality indicator.

On the other hand, there is no consensus among experts on standards for rates of caesareans that reflect good quality EmOC [56]. WHO and UNICEF estimate a 5% minimal rate of caesareans among expected deliveries. The rate of major obstetric interventions (MOI) carried out for absolute maternal indications (AMI) would be a more precise indicator, but is rarely used. It should vary between 1% and 2% of expected deliveries. The routine data available from the information systems do not often allow this rate to be calculated. Furthermore, due to the reduced size of the study population, this indicator lacks statistical power for monitoring progress achieved in the quality of care [56].

Besides these indicators, the health of mothers was estimated by means of hospital morbidity and mortality data. For children, EmOC quality can be measured by the number of stillbirths [48], but none of the selected studies specifically addressed the results of EmOC quality for newborns.

## Discussion

In the health care sector overall, and maternal health in particular, HR are recognized as indispensable to intervention efficacy [3,11,13,65]. Despite the evidence, health policies are slow to give HR their due [13,65,66]. This review confirms the importance of HR in EmOC services; an HR component is readily identified and fundamental in every aspect of EmOC quality. Nevertheless, the level of available evidence varies markedly depending on which dimension of quality is considered. The structure and results dimensions are largely documented, while processes are documented primarily from the perspective of users' satisfaction, but much less so with respect to the technical aspects of care, even though this is a major element of the quality, and thereby ultimately the efficacy, of EmOC.

This review leads to three main conclusions: (1) staff shortages are a major obstacle to providing good-quality EmOC; (2) women are often dissatisfied with the care they receive during childbirth; and (3) the technical quality of EmOC has not been adequately studied. The first two conclusions provide lessons to consider when formulating EmOC policies, while the third point is an area where more knowledge is needed.

### Staff shortages

As reported in econometric and historical analyses, improved maternal health is linked with the density and professionalization of health personnel [11,67]; policies aimed at increasing the production of EmOC personnel have been proposed and even applied. These policies should be refined to take into account more subtle imbalances in relation to EmOC personnel. Thus, certain major constraints such as those related to gender, social class and ethnicity need to be considered, in addition to flagrant imbalances between urban and rural settings [68].

Taking these factors into account when trying to improve the availability of EmOC personnel remains a formidable challenge. Women patients, concerned about having their privacy respected, often express a preference for female personnel [43,62]. Thus the production of EmOC staff, already mostly female, should be increased, especially in rural settings. Yet, whether for family reasons or because of instability in certain regions, women are more reluctant to be assigned to rural areas [34,42].

Moreover, the use of female staff is often associated with higher rates of absenteeism [43,68,69]. Therefore, the production of personnel needs to be combined with measures to attract and retain staff in rural areas but should also include the best HR management strategies to limit productivity losses due to absences. In addition, having personnel from the same social class and ethnicity as the population being served would lower the social barriers to communication between staff and patient [60] and should therefore also be considered.

While still trying to address EmOC staff shortages, the quantitative objectives of health policies should be revisited, updated and adapted to changing contexts. Most studies continue to refer to the original WHO standards, for which the basis of calculation is now being questioned.

New standards are estimated at 20 midwives, or equivalent staff, and health centres of 60 to 80 beds in a district of 120 0000 inhabitants. This staff distribution would depend on the population's dispersal: either nine or 10 midwives in a hospital, and the rest in the health centres of the district, or one midwife per village, with intensification of the referral system [3]. These new standards, although better adapted because they take into account population size, needs and existing health structures, are nevertheless still based on a normative approach, and the validity of these normative references is being called into question [3,70].

New approaches are probably more indicated for estimating and correcting staff shortages in EmOC. One such approach is the WISN (Workload Indicators of Staffing Need). This approach, by estimating the ratio between the current and desired workload level by type of personnel, supports the formulation of specific recommendations for staff deployment in each health facility. While its validity depends on the quality of the routine administrative data collected, it nevertheless helps to restrain both overstaffing and understaffing [71].

Policies aimed at redressing EmOC staff shortages should be developed in tandem with initiatives to improve the qualifications and skills of EmOC personnel. As in the transition to qualified birth attendance [7], clinical teams that combine diverse skills will maximize EmOC efficacy and coverage. The best configurations for creating cost-effective EmOC teams that would be acceptable to both staff and patients remain to be defined [7,33].

While some countries might be able to justify using less-qualified EmOC personnel, it is not known whether their effectiveness could make up for increased supervision requirements, and whether this personnel would be acceptable to the population and to professional associations [7,11,33]. We should not forget that the failure of the policy regarding traditional birth attendants is due mainly to the lack of technical legitimacy in their training, the excessive supervision required, the difficulty of adapting their training to the great diversity of delivery situations, and to interprofessional conflicts generated by this policy [3,11].

Despite these uncertainties, the range of competences of EmOC teams should extend to the ability to manage the care of newborns in general, and those of seropositive mothers in particular, given the increasing magnitude of this problem. Moreover, policies concerning EmOC personnel should extend beyond the restrictive definition of technical staff (physicians, midwives, etc.) to include administrative and support staff, without whom many EmOC interventions would fail [48,56].

### Patient satisfaction

General dissatisfaction with the interpersonal quality of EmOC is often reported. Some have suggested modifying the curricula of EmOC personnel to address these complaints. Others call into question certain technical acts (such as gynaecological positioning) and encourage research inspired by women's traditional practices to increase acceptability [57-59,63]. The impacts of such recommendations may be a long time in coming.

In fact, considerable time will be required before the generation of EmOC personnel trained under the new curriculum is functional and before the results of research are validated and activated. Meanwhile, if nothing is done, patient dissatisfaction could result in even lower attendance at health facilities, thereby reducing EmOC coverage and the rate of hospital-based deliveries, and generally slowing any progress in maternal health [7,57,58,63]. An intermediate solution could be to introduce some patient-focused communication systems, using the personnel currently in place to encourage a mediation of cultures among patients and caregivers [60].

### Technical quality of EmOC

This review shows that data for evaluating the technical quality of EmOC are scarce. Variations in quality are linked with rates of maternal mortality that differ significantly among countries with comparable technical, financial and human inputs [72]. Moreover, these variations make it possible to discriminate among countries that make progress in reducing maternal mortality. They should therefore be analysed, and at the centre of these variations in quality is EmOC staff performance [45,71].

Other studies should analyse staff performance in greater depth, and particularly their executive competence, which is scarcely documented in the selected studies. Bearing in mind, on the one hand, ethical and logistical constraints, and on the other, the extent to which staff performance depends on material and organizational resources [47,48], existing theoretical models and robust research designs should, with valid instruments, make it possible to evaluate and analyse the executive competence of EmOC personnel.

Some other aspects of EmOC staff performance that have rarely been examined, such as organizational stability and staff productivity, should also be analysed [14,15,72]. Because EmOC services are good markers of health system performance, the analysis of staff performance, in terms of organizational stability, could help to orient other priority health interventions. Organization stability involves using staff so as to guarantee the viability of services and their capacity to adapt to change [16]. Analysing and improving staff productivity could generate important productivity gains through effective time management of staff currently in place [14,15].

As a published literature-based study, this review could be subject to a publication bias; selected studies are identified in computerized databases, while unpublished studies, grey literature, books and monographs are missed. Moreover, as in any research based on keywords, the generic aspect of the word choice may lead to certain studies' being ignored. Finally, for results evaluating the quality of care of the newborn child, the use of keywords such as "stillbirth" would probably have allowed us to find more relevant works.

## Conclusion

Human resources are the key component in all the dimensions of EmOC services and determine their quality, particularly in clinical processes. This review demonstrates that there are robust data on the negative impacts of staff shortages and of certain qualitative imbalances, such as in gender or social class, on the production of good-quality EmOC. Taking patients' preferences regarding the clinical setting and the attitudes of the clinical staff into consideration would help to improve access to and utilization of EmOC.

Remedial policies to address staff shortages are being developed and implemented, but they will be even more effective if they take into account these more qualitative aspects. These policies should aim to correct quantitative imbalances, introduce measures to retain female staff in rural settings and respect users' preferences. This last point is a major challenge that must be undertaken both in a long-term perspective, through curriculum change, as well as in the short term by encouraging innovations in existing systems. These policies must be implemented with the full involvement of EmOC personnel, broadly defined, as suggested in this review, using an integrated and multisectoral approach. In this way, the performance of health systems will be very tangibly improved.

Paradoxically, the processes of producing good-quality services are less well documented, even though they are fundamental to the services' effectiveness. Because the structural deficiencies are so great, analyses have tended to focus on them. Yet variations in quality account for important differences in outcomes. These processes must be better documented in order to promote high quality services.

By updating EmOC human resources policies and better understanding the mechanisms of production of quality services in disadvantaged settings, the stage will be set for EmOC services to fully assume their role and contribute significantly to reducing maternal and infant mortality and, thereby, to achieving the fourth and fifth Millenium Development Goals.

## Competing interests

The authors declare that they have no competing interests.
